# Characterization of the relationship between dose and blood eosinophil response following subcutaneous administration of mepolizumab 

**DOI:** 10.5414/CP202446

**Published:** 2015-10-07

**Authors:** Isabelle J. Pouliquen, Oliver Kornmann, Sharon V. Barton, Jeffrey A. Price, Hector G. Ortega

**Affiliations:** 1 Clinical Pharmacology Modeling and Simulation, GlaxoSmithKline, Uxbridge, UK,; 2 IKF Pneumologie Frankfurt, Clinical Research Centre Respiratory Diseases, Frankfurt, Germany,; 3 Clinical Statistics, GlaxoSmithKline, Stevenage, UK,; 4 Clinical Unit Cambridge, Addenbrookes Hospital, GlaxoSmithKline, Cambridge, UK, and; 5 Respiratory Therapeutic Area Unit, Research & Development, GlaxoSmithKline, Research Triangle Park, NC, USA

**Keywords:** blood eosinophils, monoclonal antibody, mepolizumab, asthma, IL-5, pharmacokinetics, pharmacodynamics, safety, subcutaneous, absolute bioavailability

## Abstract

Objective: Mepolizumab is a humanized IgG1 monoclonal antibody that blocks human IL-5 from binding to the IL-5 receptor, which is mainly expressed on eosinophils. Eosinophils are key cells in the inflammatory cascade of various diseases, including asthma. This study investigated the pharmacokinetic (PK)/pharmacodynamic (PD) relationship between exposure of mepolizumab subcutaneous (SC) administration and blood eosinophil reduction compared with intravenous (IV) administration in adult subjects with asthma. Methods: In this multi-center, randomized, open-label, parallel-group, repeat-dose study, 70 adult subjects received one of four possible treatment regimens: mepolizumab 12.5, 125, or 250 mg SC or 75 mg IV. In addition to analyzing the dose and PK/PD relationship, absolute bioavailability, safety, tolerability, and incidence of anti-mepolizumab antibodies were evaluated. Results: Blood eosinophil levels decreased in a dose-dependent manner with the lowest (12.5 mg) dose clearly differentiating from the other doses. A non-linear inhibition Imax model based on blood eosinophil levels at week 12 identified that the SC doses providing 50% and 90% of maximal blood eosinophil inhibition were 11 mg (95% confidence interval (CI): 5.19 – 16.85) and 99 mg (95% CI: 47 – 152), respectively. The route of administration did not affect the exposure-response relationship. The estimated mepolizumab SC absolute bioavailability (arm) was 74% (90% CI: 54 – 102%). The safety profile of mepolizumab was favorable. Conclusions: A dose-dependent reduction in blood eosinophils across all mepolizumab doses investigated was observed. The subcutaneous absolute bioavailability was 74%. The route of administration did not affect the mepolizumab exposure eosinophil response relationship.

## Introduction 

Overexpression of IL-5 has been reported in patients with a variety of eosinophil-associated disorders [1, 2]. IL-5 regulates multiple major eosinophil functions, including cellular proliferation, mobilization from the bone marrow into the peripheral circulation, maturation, activation, tissue recruitment, survival, and priming to stimulating agents [3, 4, 5]. Eosinophils are recruited from the circulation to sites of inflammation, where they affect the immune response through a variety of mechanisms [3, 6]. Eosinophils are involved in the initiation and propagation of diverse inflammatory responses and are a characteristic feature of various diseases such as asthma, atopic dermatitis, and allergic rhinitis. Infiltration of the bronchial epithelium and submucosa with eosinophils in asthma is a hallmark of the disease [7]. Treatment strategies aimed at reducing eosinophilic airway inflammation have led to better control of the disease and to a reduction in the number of asthma exacerbations [8, 9, 10]. 

Mepolizumab is a humanized IgG1 monoclonal antibody that blocks human IL-5 from binding to the IL-5 receptor that is mainly expressed on eosinophils but is also present on basophils [11]. Clinical studies have shown that mepolizumab is an effective treatment that reduces the risk of asthma exacerbations in patients with severe eosinophilic asthma [9, 10]. Following administration of mepolizumab, a decrease in blood eosinophils has been consistently observed in patients with various eosinophilic conditions and in healthy subjects [9, 10, 12, 13, 14, 15, 16, 17, 18, 19, 20] and can therefore be used as a pharmacodynamic endpoint for mepolizumab intervention. Sputum eosinophil reduction has also been reported in patients with severe asthma after treatment with mepolizumab [9, 10, 14]. Previous studies were conducted with mepolizumab administered intravenously (IV); however, the subcutaneous (SC) route of administration is generally preferred by patients and healthcare providers. 

The use of modeling and simulation, particularly pharmacokinetic (PK)/pharmacodynamic (PD) modeling, to support the design of clinical trials has been encouraged for many years [21]. Modeling and simulation was used to describe the PK/PD relationship between mepolizumab plasma concentration and blood eosinophil levels after IV administration from previous studies and the model was extended to predict SC response. Then, a range of doses were simulated using trial simulation methods in order to optimize the likelihood of demonstrating a dose response in the study described here. 

A dose-ranging study including both IV and SC routes of administration was conducted in adult subjects with asthma and blood eosinophil levels > 300 cells/µL. The primary objective of this study was to demonstrate that the PK/PD relationship between exposure of mepolizumab administered SC and blood eosinophil reduction (a marker of clinical response), is unchanged compared with IV administration. In addition, the absolute bioavailability of the SC route of administration, the levels of anti-mepolizumab antibodies and the safety and tolerability of mepolizumab were assessed. 

## Methods 

### Study design 

**Figure S1.**
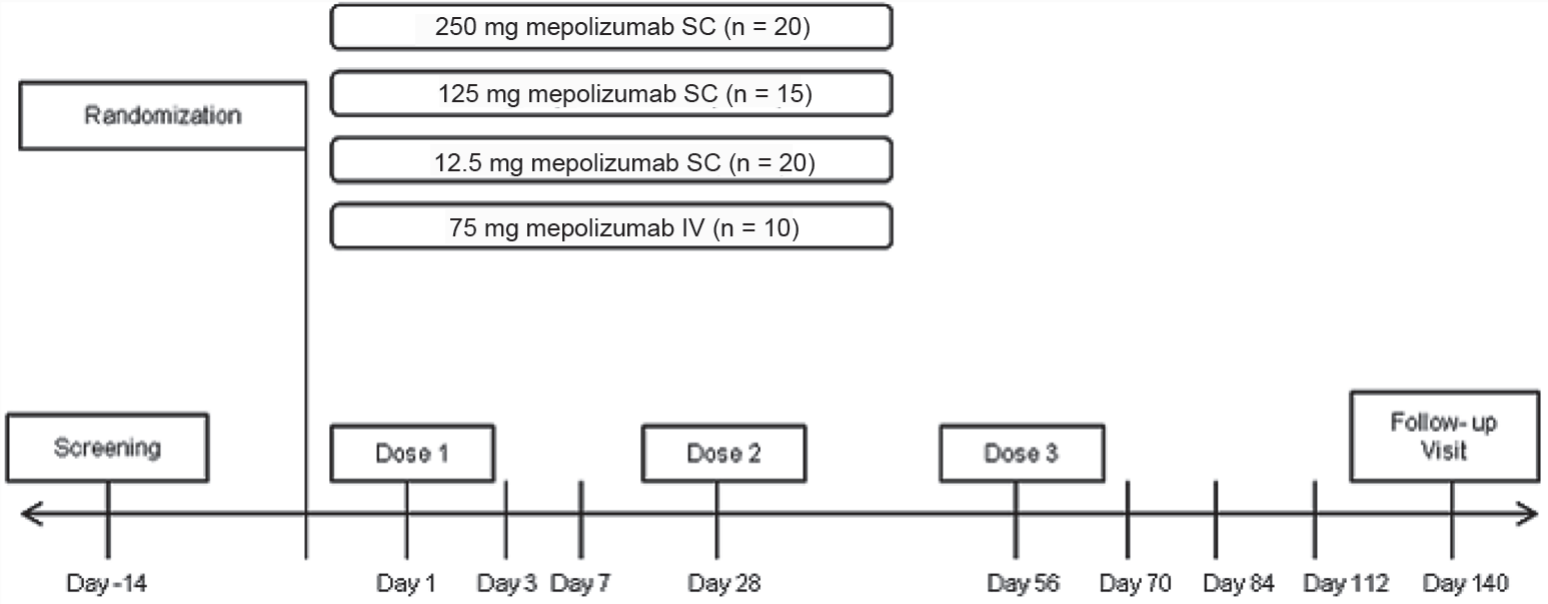
Study design schematic.

This study was a multicenter, randomized, open-label, parallel-group, repeat-dose phase IIa study in adult subjects with asthma and blood eosinophils > 300 cells/µL (clinicaltrials.gov identifier NCT01366521 [http://clinicaltrials.gov/ct2/show/NCT01366521]). Three SC doses 12.5, 125, and 250 mg and a 75 mg IV dose administered every 4 weeks (q4w) for 12 weeks were investigated. Subjects were evaluated up to day 140 (see Supplemental material for a study design schematic, supporting Figure S1). 

From an initial PKPD model developed based on previous data, dose response curves were simulated and the lowest and highest SC doses of 12.5 and 250 mg were selected to be at or below the predicted dose associated with 50% of the maximal inhibition effect attributable to drug (ID_50_) and to fall at the top of the blood eosinophil reduction dose-response curve, respectively. A dose of 75 mg IV, which was also investigated in a dose-ranging phase IIB study, was selected to assess the absolute bioavailability of mepolizumab and to bridge the IV PD data across studies. 

Sample size selection of 20, 15, and 20 for the 12.5, 125, and 250 mg mepolizumab SC doses, respectively, was based on simulations in excess of 500 clinical trials. The selected design was estimated to have at least 90% power to detect a dose response. A smaller sample size of 10 for the 75 mg IV cohort was selected considering PK and PD data were already available at that dose and in this patient population. Subjects were randomly assigned to one of the four possible regimens on an allocation ratio of 4 : 3 : 4 : 2 (12.5 mg SC, 125 mg SC, 250 mg SC, and 75 mg IV) in accordance with a computer-generated randomization schedule using an internal validated system. 

The study was conducted at 11 centers in 4 countries (Estonia, France, Germany, and USA) in accordance with Good Clinical Practice, all applicable regulatory requirements and the guiding principles of the Declaration of Helsinki. The study protocol and informed consent form were reviewed and approved by an ethics committee or institutional review board and written informed consent was obtained from each subject prior to the performance of any study-specific procedures. (Further details can be found in the Supplemental material). 

### Study population 

Eligible subjects were males or females (non-pregnant or lactating and using acceptable method of birth control if relevant) between 18 and 65 years of age, with a diagnosis of asthma for at least 1 year, receiving a stable dose of an inhaled corticosteroid (ICS) or combination therapy (ICS and long-acting β-agonist) for at least 12 weeks prior to screening. Subjects had a forced expiratory volume in 1 second (FEV_1_) ≥ 45% and < 90% of predicted normal value during screening; demonstrated airway reversibility (change in FEV_1_ ≥ 12%) within 30 minutes of inhalation of albuterol/salbutamol or airway hyper-responsiveness with a 20% fall in FEV_1_ to methacholine or histamine documented in the 12 months prior to randomization; a documented evidence of blood eosinophil levels > 300 cells/µL or ≥ 200 cells/µL in 4 subjects (after protocol amendment) that was related to asthma (subjects with a parasitic infestation within 6 months of screening were excluded). Use of albuterol/salbutamol as rescue medication on an as-needed basis was allowed during the study. (Further details can be found in the Supplemental material). 

### Study assessments 

**Timing of assessment**

Blood eosinophils were obtained as part of the standard hematologic assessments at screening and days 1 (pre-dose), 3, 7, 28 (pre-dose), 56 (pre-dose), 70, 84, 112, and 140. Blood samples for analysis of mepolizumab plasma concentrations were collected in all subjects using a sparse PK sampling scheme. Up to 18 scheduled blood samples per subject were collected on dosing days (days 1, 28, and 56) at pre-dose and 0.5, 1, and 2 hours post-dose (time was relative to the end of infusion in the IV cohort) as well as on days 3, 7, 70, 84, 112, and 140 (follow-up visit). 

To assess the presence of anti-mepolizumab antibodies, serum samples were collected pre-dose and at days 112 and 140. Induced sputum samples were obtained (where possible) at pre-dose and at days 7, 28 (pre-dose), 56 (pre-dose), and 84. Serum free and total IL-5 samples were obtained pre-dose and at days 3, 7, 28 (pre-dose), 56 (pre-dose), 70, 84, 112, and 140. Safety and tolerability were evaluated throughout the study by assessment of adverse events (AEs), vital signs measurements, electrocardiograms, and standard clinical laboratory tests. 

**Analytical methods**

Plasma concentrations of mepolizumab were determined using two validated bioanalytical immunoassay methods, both with a Lower Limit of Quantification at 50 ng/mL. Mepolizumab plasma concentrations were quantified by either using an immunoassay method with a biotinylated recombinant human IL-5 bound to a streptavidin coated microtiter plate or a neutralizing idiotypic antibody, specific for the binding portion of mepolizumab, passively adsorbed to a polystyrene microtiter plate as the means of capture. Mepolizumab was then detected using an Fc specific mouse anti-human IgG1 labeled with horseradish peroxidase with a chemiluminescent endpoint. 

The anti-drug antibodies (ADA) assay (screening, confirmation, and titration analysis) for the presence of anti-mepolizumab antibodies analysis was validated according to published recommendations [22, 23]. The ADAs were detected using a Meso Scale Discovery (MSD) Electrochemiluminescent bridging assay. Induced sputum samples were processed within 2 hours of the end of the induction procedure at the investigator’s site, as previously described [10]. The prepared slides were sent to a centralized laboratory (Glenfield Hospital, Leicester, UK) for differential cell count analyses. 

Serum free and total IL-5 were both measured by fully validated “fit-for-purpose” methods using an electrochemiluminescence-based immunoassay on the MSD platform. The quantifiable ranges for the total IL-5 assay were 7.81 – 500 pg/mL and for the free IL-5 assay 3.91 – 500 pg/mL. Spirometry assessments met the American Thoracic Society/European Respiratory Society standards [24]. Additional information on the analytical methods can be found in the Supplemental material. 

**Statistical analysis**

*Pharmacokinetics*

The PK population was defined as all subjects randomized to treatment who received at least one dose of study treatment and who had at least one PK sample taken and analyzed. 

Population modeling techniques using nonlinear mixed effects methods (NONMEM 7, ICON Development Solutions, Ellicott City, MD, USA) were used to estimate individual and population mepolizumab PK parameters. Plasma mepolizumab concentrations from the SC and IV cohorts were modeled independently using a population PK model developed based on previous mepolizumab data from healthy volunteers and subjects with asthma. 

A two-compartment model with first order elimination and a two-compartment model with first order absorption and first order elimination were selected for the IV and SC data respectively, based on prior knowledge of mepolizumab PK (further details can be found in the Supplemental material). Covariates were investigated but amongst the covariates available (sex, bodyweight, age, baseline blood and sputum eosinophils, baseline free and total IL-5 and baseline inhaled corticosteroids) only body-weight on plasma clearance (CL or CL/F) and volume of the central compartment or apparent volume of the central compartment were found to be statistically significant and retained in the final models. Allometric scaling was used with regression coefficient fixed to 0.75 for clearance and unity for volume of the central compartment; bodyweight was centered to 70 kg. The final model was evaluated using goodness of fit plots. Upon model building completion, individual post-hoc PK parameter estimates were obtained. 

The absolute bioavailability of the SC route of administration based on the area under the plasma concentration-time curve (AUC) was derived from the individual post-hoc clearance estimates obtained after SC and IV administration. The ratios of dose-normalized maximum observed plasma concentration (C_max_) between the SC and IV routes of administration after the first and third dose were also derived from the individual C_max_ estimates obtained after SC and IV administration. Log-transformed clearance and log-transformed dose-normalized C_max_ were analyzed using analysis of variance models. 

*Pharmacokinetics/pharmacodynamics*

Blood eosinophil count vs. plasma mepolizumab concentrations data were modeled using an indirect response population PKPD model, with Hill slope fixed to unity, developed based on data from earlier studies (further details can be found in the Supplemental material). Modeling occurred in two steps. Firstly, individual predicted mepolizumab plasma concentrations at the time of the blood eosinophil measurements were obtained from the IV and SC population PK models and then merged with the blood eosinophil data. Secondly, these data (IV and SC together) were modelled. Covariates were investigated but amongst the covariates available (same as in the population PK model) only baseline blood eosinophil counts on the predicted baseline were found to be statistically significant and retained in the final model. The final model was evaluated using goodness of fit plots. To test the impact of the route of administration on the PKPD relationship, an additional term (of the form 1+(α×I) where I is an indicator variable for the route of administration) on the concentration inducing 50% of maximal inhibitory effect (IC_50_) was added to the model and the objective function (OF) with and without this parameter compared. 

*Pharmacodynamics*

The PD population was defined as all subjects randomized to treatment who received at least one dose of study medication and who also had a baseline PD measurement and at least one post-treatment PD measurement. 

Blood and sputum eosinophil derived PD parameters were calculated and summarized using descriptive statistics (further details can be found in the Supplemental material). Note, blood eosinophil count is expressed in cells/µL in the text but in GI/L in the tables and figures. 

*Blood eosinophils dose response*

A non-linear 3-parameter inhibition Imax dose-response model was fitted to the change from baseline in log10-transformed blood eosinophil levels at week 12 (day 84), incorporating all doses. Baseline log10-transformed blood eosinophil count was included in the model as a covariate effect on the intercept. Response at zero dose was assumed to be zero. 

The absolute bioavailability of the SC route of administration was assumed to be 75% for the purposes of the dose-response modeling based on previous trial data and confirmed within this trial. The 75 mg IV dose was therefore considered to equate to 100 mg SC. Model parameter estimates and 95% confidence intervals (CIs) were presented. Estimates and 95% CIs for the minimal proportion of baseline blood eosinophils remaining at week 12 (accounting for the mean baseline blood eosinophils across dose groups) and for the SC dose providing 90% of the maximal inhibition of blood eosinophils attributable to the drug at week 12 were also calculated post hoc. Analyses were conducted using SAS (versions 9.1.3 and 9.2; SAS Institute, Cary, NC, USA). 

Total and free IL-5 data were summarized using descriptive statistics. 

## Results 

### Study subjects 

**Figure 1.**
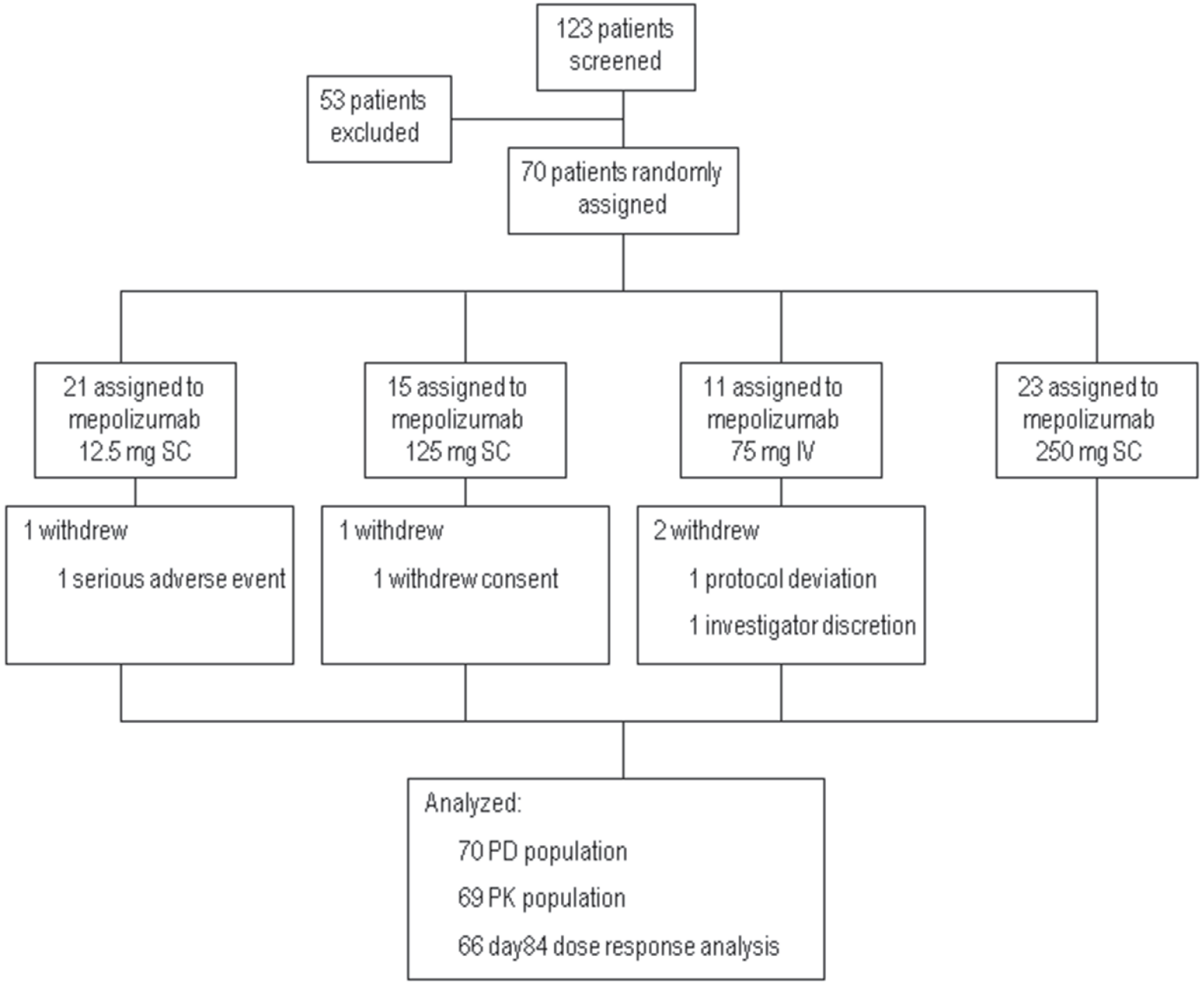
Subject disposition.

A total of 70 subjects were enrolled, of which 66 subjects completed the study: one subject withdrew consent; one withdrew due to a protocol deviation (failed exclusion criterion); one withdrew due to a serious adverse event (SAE) (bladder papilloma) considered by the investigator to be unrelated to mepolizumab; and one withdrew at the investigator’s discretion (subject left the country and would not return before the follow-up visit) (Figure 1). 

Subject demographics and baseline disease characteristics including pulmonary function were overall comparable between the treatment groups (Table 1); with the exception of weight (in the 75 mg IV group) and a slight imbalance in blood and sputum eosinophils across groups (slightly lower in the SC 125 mg group and in particular in the IV 75 mg group compared with the SC 12.5 mg and 250 mg groups). 

### Pharmacokinetics 

**Figure 2.**
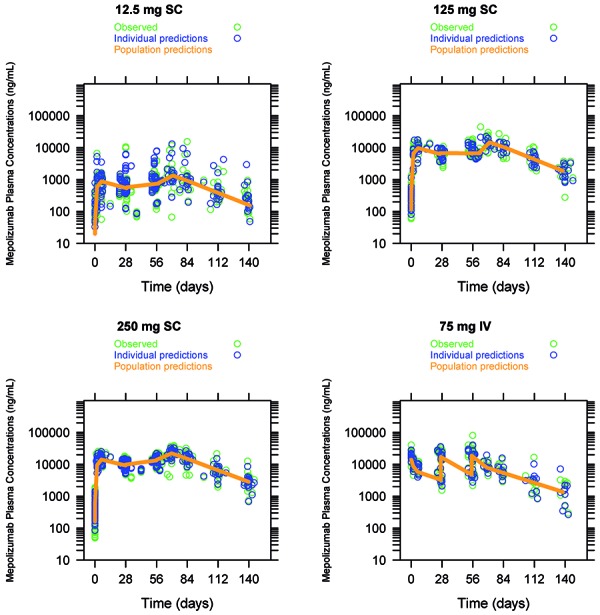
Individual mepolizumab model-predicted and observed plasma concentration-time profiles with population prediction.

Mepolizumab population PK parameter estimates and the predicted and observed mepolizumab concentration-time course are presented in Table 2 and Figure 2. After repeat IV or SC administration (three doses), mepolizumab clearance (CL) or apparent clearance (CL/F) was slow; the volume of distribution at steady state or apparent volume of distribution at steady state, inclusive of both the central (Vc) and peripheral (Vp) compartments, was approximately equal to the plasma volume plus the interstitial space, suggesting that there is limited drug distribution into the tissues. The terminal half-life derived from the individual post-hoc PK parameter estimates was ~ 22 and 28 days following SC and IV administration, respectively. 

Median time to maximal concentration (t_max_) was 6 – 8 days post-dosing in the SC dose groups compared with 0.5 hours in the IV group. CL/F and V/F were dose independent. After three SC administrations q4w, a 1.7 accumulation ratio was estimated for the AUC over the dosing interval (AUC_(0-τ)_) and C_max_, as expected for q4w dosing. For all SC cohorts combined the estimated absolute bioavailability was 74% (90% CI: 54 – 102%; p = 0.1222). The PK profiles in the 12.5 mg SC group were more variable than in the other groups. 

The estimated dose-normalized C_max_ ratio (SC/IV) after the first and third dose (all SC dose combined) administered was 42% (90% CI: 30 – 58%) and 54% (90% CI: 39 – 74%), respectively. 

### Pharmacokinetics/pharmacodynamics 

**Figure 3.**
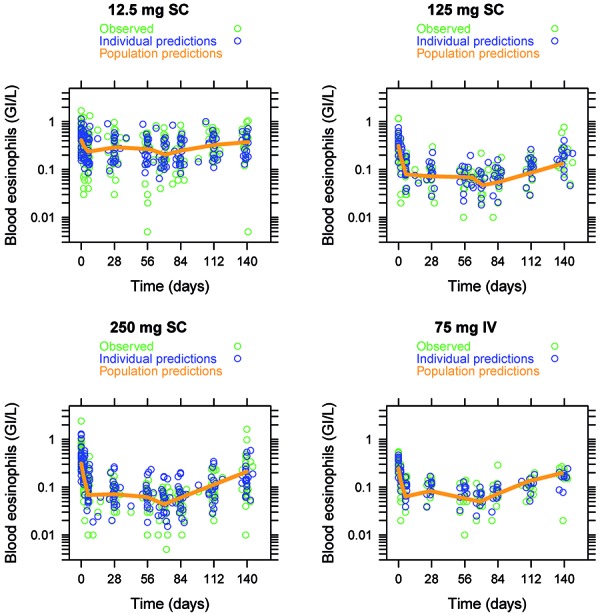
Individual model-predicted and observed blood eosinophils-time profiles with population prediction.

After repeat IV and SC administration, there was a clear relationship between blood eosinophil counts and mepolizumab plasma concentrations. This relationship was well described by an indirect response model. Mepolizumab population PD parameter estimates obtained from this model and the predicted and observed blood eosinophil-time course are presented in Table 3 and Figure 3. In order to test the impact of the route of administration, an additional parameter on IC_50_ was incorporated and the OF with and without this parameter compared. The decrease in the OF was < 3.84 for the model including the additional parameter on IC_50_ (equivalent to p > 0.05) and therefore was not statistically significant. 

### Blood eosinophils 

**Figure 4.**
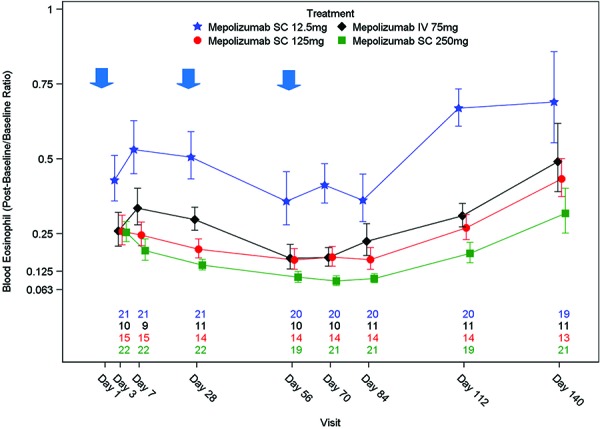
Proportional change from baseline in blood eosinophil data (Geometric mean ± standard error). Blue arrows indicate mepolizumab administration. N’s represent number of patients within each treatment group with available data at each time point. The order from the top is Mepolizumab SC 12.5 mg, Mepolizumab IV 75 mg, Mepolizumab SC 125 mg, Mepolizumab SC 250 mg. A proportional change from baseline of < 1 represents a reduction in eosinophil levels. A proportional change from baseline of 1 represents no change in eosinophil levels. Prior to log-transformation, zero values were imputed with half the minimum value across all dose groups and time points.

Mepolizumab administered q4w produced a dose- and time-dependent reduction in blood eosinophils (Figure 4). Levels of blood eosinophils decreased from baseline (pre-dose on day 1) in all four treatment groups. A pronounced reduction was seen by day 3 (first post-dose measurement) in all groups but to a lesser extent in the 12.5 mg group. Further marginal reductions were observed between day 7 and day 84 (end of treatment period) in all groups. Post-baseline blood eosinophil counts were approximately in the same range for the mepolizumab SC 250 mg, SC 125 mg, and IV 75 mg groups, while higher counts were observed in the mepolizumab SC 12.5 mg group. Blood eosinophils began to return to baseline from day 70 (2 weeks post-last dose) or day 84 (4 weeks post-last dose) up to day 140 (follow-up) in all groups. Blood eosinophils measured at day 140 had not completely returned to baseline levels. Derived blood eosinophil parameters are presented in Table 4. 

### Blood eosinophil dose response analysis 

**Figure 5.**
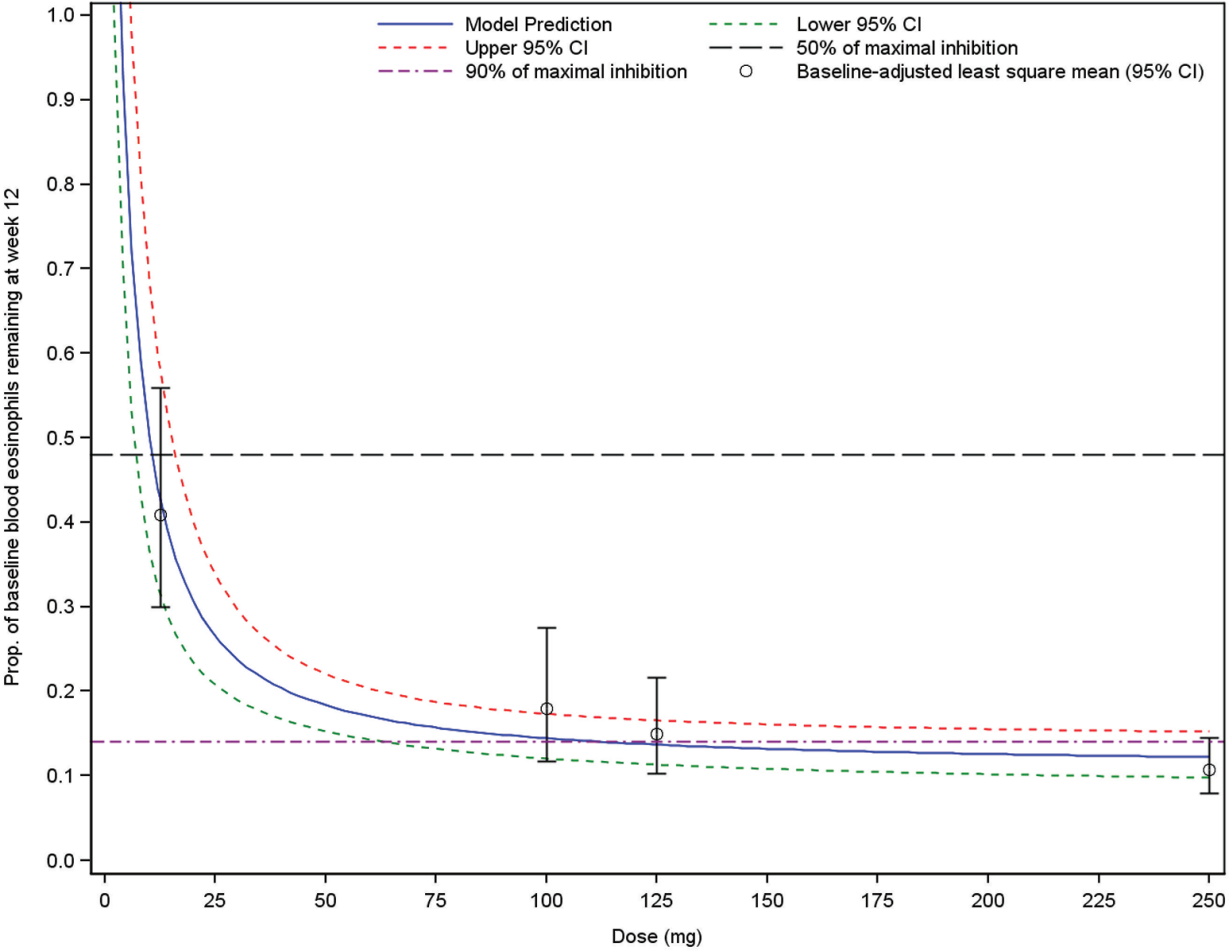
Proportion of baseline blood eosinophils remaining at week 12 (day 84) (model predictions and 95% CIs) non-linear Imax dose-response model). 10^R^ = 10^β(Bl) + (D^^×^^Imax)/(D^^+^^ID50). ^ R = Change from baseline in log_10_-transformed blood eosinophil levels (week 12); D = Dose (mg) (SC or SC dose equivalent); Bl = Baseline log_10_ blood eosinophils (mean across all dose groups = –0.36); β = baseline covariate regression coefficient (estimate: –0.84; 95% CI: (–1.14 – –0.55)); Imax = Maximum reduction in log_10_ blood eosinophils (week 12) (estimate: –1.27; 95% CI: (–1.43 to –1.11)); ID_50_ = Dose inducing half maximal drug effect (estimate: 11.02; 95% CI: (5.19 – 16.85)); 75 mg IV assumed to equate to 100 mg SC within model. Prior to log_10_-transformation, zero values were imputed with half the minimum value across all dose groups and time points.

A dose-response relationship for the change from baseline in log10-transformed blood eosinophil counts at week 12 (day 84) was observed. Based on a non-linear inhibition Imax model, the proportions of baseline blood eosinophils remaining at week 12 were comparable in the 75 mg IV, 125 mg SC, and 250 mg SC groups (0.14, 0.14, and 0.12, respectively). In contrast, the proportion remaining at week 12 in the 12.5 mg SC group was much higher (0.43). The SC doses estimated to provide 50% and 90% of the maximal inhibition of blood eosinophils attributable to the drug at week 12 were 11 mg (95% CI: 5 – 17) and 99 mg (95% CI: 47 – 152), respectively. The estimated minimal proportion of baseline blood eosinophils remaining at week 12 accounting for the mean baseline blood eosinophils across dose groups was 0.11 (95% CI: 0.08 – 0.14) (Figure 5). 

### Sputum eosinophils 

**Figure 6.**
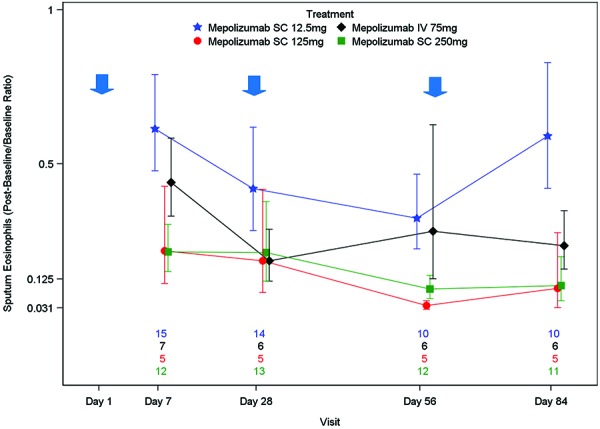
Proportional change from baseline in sputum eosinophil data (%) (Geometric mean ± standard error). Blue arrows indicate mepolizumab administration. N’s represent number of patients within each treatment group with available data at each timepoint. The order from the top is Mepolizumab SC 12.5 mg, Mepolizumab IV 75 mg, Mepolizumab SC 125 mg, Mepolizumab SC 250 mg. A proportional change from baseline of < 1 represents a reduction in eosinophil levels. A proportional change from baseline of 1 represents no change in eosinophil levels.

Sputum eosinophils also decreased from baseline (pre-dose on day 1) in a dose-dependent manner (Figure 6). This is also reflected in the derived sputum eosinophil parameters (Table 4). Reduction was observed from day 7 (first post-dose measurement) to day 84 (last post-dose measurement) in all groups. The largest decrease from baseline was observed in the mepolizumab SC 125 mg and 250 mg groups, with less of a decrease in the mepolizumab SC 12.5 mg group. Sputum eosinophils appeared to return towards baseline by day 84 in the SC 12.5 mg group, though by day 84 they had not returned to baseline levels. It is worth noting that the number of subjects who provided sputum data was small and the data were variable. No statistical analysis was conducted. 

### Total and free IL5 

**Figure 7.**
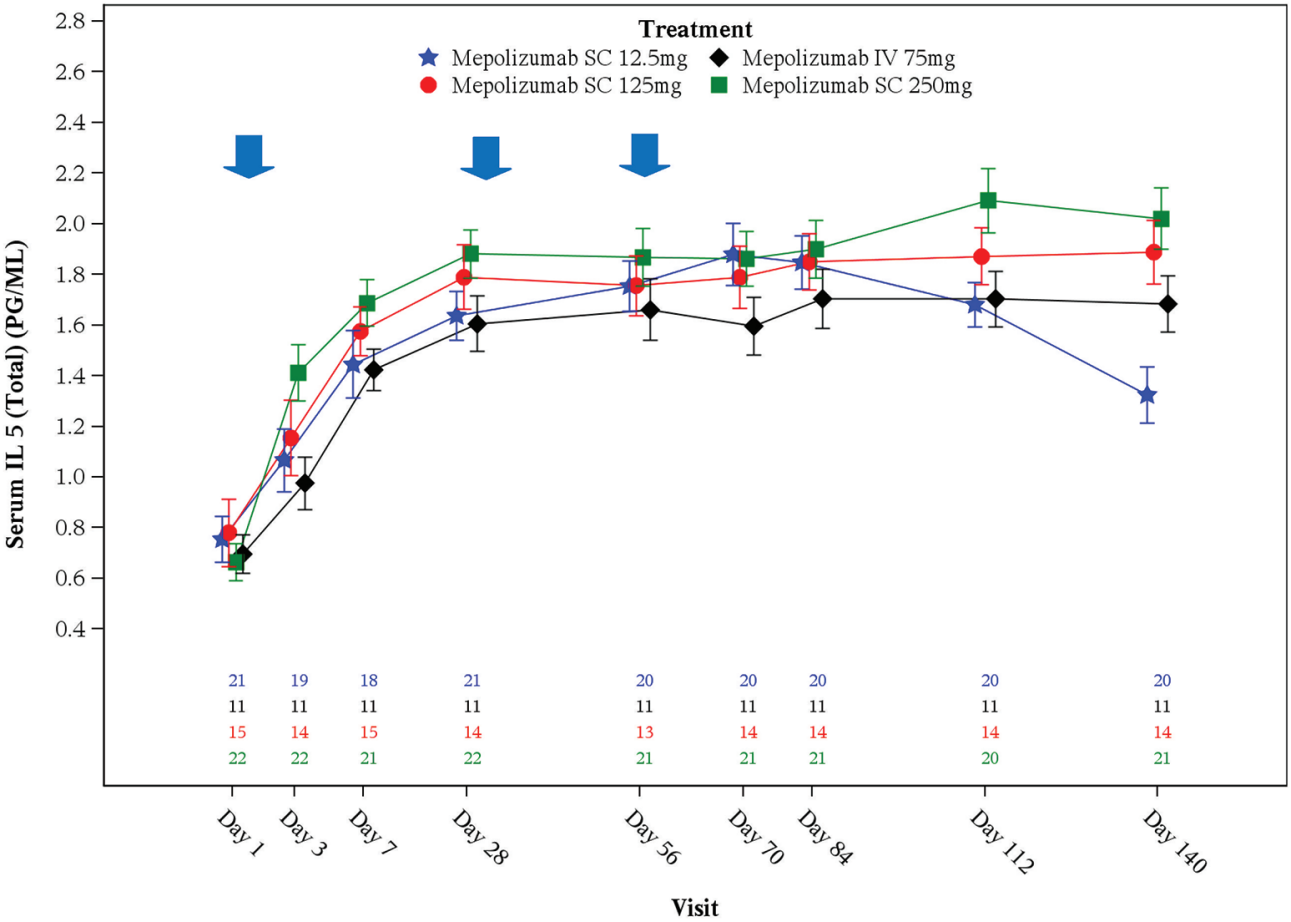
Serum IL-5 (total) data (log10 scale) (mean ± standard error). Blue arrows indicate mepolizumab administration. N’s represent number of patients within each treatment group with available data at each time point. The order from the top is Mepolizumab SC 12.5 mg, Mepolizumab IV 75 mg, Mepolizumab SC 125 mg, Mepolizumab SC 250 mg; Results below the limit of quantification have been imputed with half the lower limit of quantification.

Serum free and total IL-5 concentrations were non-measurable at baseline in most subjects (94% and 89%, respectively) (Table 1). Following treatment with mepolizumab mean serum total IL-5 levels increased with similar magnitude from baseline across all doses (in all but two subjects) up to day 28 and then remained constant up to day 140 in all treatment groups except 12.5 mg SC. In the 12.5 mg SC cohort, after day 84 a decrease in serum total IL-5 levels was observed although levels did not return to baseline by day 140 (Figure 7). Exploratory plots showed no relationship between serum total IL-5 and blood eosinophils or between levels of serum total IL-5 and mepolizumab plasma concentrations. 

###  Safety 

Overall, the safety profile of mepolizumab was favorable; most AEs were reported as mild or moderate in intensity. The percentage of subjects reporting AEs after SC (56%) and IV (55%) dosing was similar (see Supplemental material, supporting Table S1). No fatalities were reported; only one SAE of bladder papilloma was reported in the 12.5 mg mepolizumab SC group. No signs or symptoms associated with exaggerated response (rebound) were observed during the follow-up period after stopping mepolizumab treatment. The most frequently reported AE and drug-related AE was injection site reaction (further details can be found in the Supplemental material). 

### Immunogenicity 

The presence of ADAs was detected in 8 subjects out of 70 (11%) and in 13 samples (two pre-dose, six at day 112, five at follow-up) out of 201 (6%). These subjects were distributed across the three SC groups. No ADAs were detected in the 75 mg IV group. Of the 2 subjects with a positive pre-dose sample, 1 had no positive samples post-dose and one had positive samples at both day 112 and follow-up. 

In these subjects who had positive samples there was no correlation between the presence of ADAs and AEs and no apparent marked changes in the PK or blood eosinophil profiles. All samples were negative for neutralizing antibodies. 

## Discussion 

The primary objective of the study was to demonstrate that the exposure-response relationship for mepolizumab via the SC route of administration was unchanged compared with the IV route. To achieve this and to allow adequate characterization of the dose-response relationship for blood eosinophil reduction following SC administration: i) subjects with eosinophilic asthma were enrolled; ii) a 75 mg IV dose of mepolizumab (as a reference); iii) an expected sub-therapeutic SC dose of 12.5 mg as well as 125 mg and 250 mg SC doses were included in the study. Predictions from simulations showed that the selected SC low dose (12.5 mg SC) was anticipated to be at or below the ID_50_ for blood eosinophil reduction, after correction for SC absolute bioavailability. 

PK parameter estimates following IV and SC administration derived using sparse PK sampling were similar to those observed in previous mepolizumab studies in subjects with asthma [15] and consistent with the PK profiles for other anti-cytokine IgG1 monoclonal antibodies [25]. Overall, mepolizumab PK was approximately dose proportional, linear and time-independent following SC administration with limited accumulation observed following administration of 3 q4w doses, which is consistent with mepolizumab half-life of ~ 3 weeks. Higher between-subject variability was observed following SC administration compared with IV which might reflect the larger sample size and the absorption process. The estimated absolute bioavailability after SC administration in the arm was 74% (90% CI: 54 – 102%), consistent with the previous value of 75% reported in healthy subjects [15]. 

Following mepolizumab administration, a pronounced reduction in blood eosinophil levels was observed in all treatment groups by day 3, with near maximal reductions already observed by day 28 demonstrating considerable reduction in blood eosinophils following a single administration. The blood eosinophil reduction observed in this study was consistent with previous mepolizumab studies [9, 10, 12, 13] conducted in patients with asthma. As anticipated, blood eosinophils decreased in a dose-dependent manner with the 12.5 mg SC dose clearly differentiating from the higher doses. 

Blood eosinophil repletion rates after treatment were also dose-dependent and slightly faster in the 12.5 mg SC group, although only a few subjects (< 10%) in the three higher dose groups had reached ≥ 50% blood eosinophil repletion by day 140, compared to 38%, in the 12.5 mg SC group. A clear relationship between blood eosinophils and mepolizumab plasma concentration was observed. This relationship was well characterized using an indirect response model and was found to not be affected by the route of administration. 

The reduction in blood eosinophils following SC administration was well described by a non-linear inhibition Imax dose-response model, unlike in the exacerbation study by Pavord et al. [10] which did not include a dose lower than 75 mg IV. From the model, the SC doses generating 50% and 90% of maximal blood eosinophils inhibition attributable to the drug at week 12 were estimated to be 11 mg and 99 mg, respectively, corresponding to IV doses of 8 mg and 74 mg, respectively, assuming an a priori value of 75% for the SC route absolute bioavailability. Thus the estimated ID_50_ was similar to the lowest dose (12.5 mg) investigated in the present study, as predicted from simulations performed prior to conducting the study. The concordance of the blood eosinophil reduction between the 75 mg IV and 125 mg SC doses further support that route of administration does not affect the mepolizumab exposure-response relationship to blood eosinophils. More importantly, the dose-response analysis identified the 75 mg IV dose (i.e., 100 mg SC) as the pharmacologic effective dose achieving 90% of the maximum effect on blood eosinophils, attributable to the drug. Consistent blood eosinophil reduction with the 75 mg IV dose was observed in the current study and other clinical studies [10, 26, 27]. 

Similarly, assessment of sputum eosinophils demonstrated a dose-related decrease in the percent eosinophils with increasing mepolizumab dose, with the 125 mg and 250 mg dose groups showing the greatest reductions. This observation was also described in a previous study [10]. A linear relationship was observed between log10-transformed blood and log10-transformed sputum eosinophils at baseline at the population level (correlation coefficient = 0.66, p < 0.0001). 

Post-mepolizumab dosing, serum total IL-5 levels (free and complex) increased from baseline in almost all subjects, suggesting that mepolizumab was bound to IL-5 in circulation as an antibody-cytokine complex, not bioactive, and demonstrating target engagement. This increase in total IL-5 is consistent with a reduction in the clearance of IL-5 due to its binding to mepolizumab. There was however no clear dose-response for the maximum total IL-5 levels achieved, implying that saturation of the target had occurred in the systemic compartment at all doses. The decrease in serum total IL-5 levels post-day 70 in the 12.5 mg SC group suggests that mepolizumab plasma concentrations were too low at the lowest dose to sustain the neutralization of IL-5. 

Serum free IL-5 was measured in this study but was non-quantifiable at baseline in most subjects (Table 1). IL-5 does not, therefore, appear to be a useful biomarker to quantify eosinophilic inflammation in this population, nor as a pharmacodynamic endpoint for evidence of pharmacology. 

## Conclusion 

In conclusion, this study shows that modeling and simulations of a relevant pharmacodynamic marker, such as blood eosinophil, and the inclusion of a wide range of doses allowed a better characterization of the dose-response relationship to support dose selection. Furthermore, a model-based rather than pairwise comparison approach allowed for prediction of responses at doses not included, but within the study dose range investigated. 

After adjusting for absolute bioavailability, the route of administration does not change the dose-response relationship. Finally, the absolute bioavailability of mepolizumab via the SC route of administration was confirmed using a sparse PK sampling scheme. 

## Acknowledgments 

Funding for this study was provided by GlaxoSmithKline, Research Triangle Park, NC (GSK study number MEA114092; clinicaltrials.gov identifier NCT01366521). All listed authors meet the criteria for authorship set forth by the International Committee for Medical Journal Editors. The authors wish to acknowledge Daren Austin, Mark Baker, Marina Bendit, Ravikant Chaubey, Ajay Daparthi, Ponmani Kanakaraj, Thomas Lee, Erik Meyer, Philip Overend, Janet Perkins, Vikram R, Abhijit Roy, Bhupesh Singh, Deborah Templeton, Kirsty Wall, Patricia Wolf, Shuying Yang and other members of the GlaxoSmithKline study team, for their contributions to the study and/or their critical review of this manuscript. The authors would also like to thank the investigators, their staff and the patients for their participation in the study. Editorial support in the form of copyediting was provided by Cheryl Wright, PhD at Gardiner-Caldwell Communications (Macclesfield, UK) and was funded by GSK. 

## Conflict of interests 

IP, SB, JP and HO are employees by GlaxoSmithKline Pharmaceuticals. 

OK has received honoraria for lectures from AstraZeneca, Boehringer Ingelheim, Novartis, Meda Pharma, for consulting from Pfizer, Novartis, for conducting clinical studies from Almirall, AstraZeneca, Bayer, Boehringer Ingelheim, Cephalon, Chiesi, GlaxoSmithKline, Mundipharma, Novartis. 

## Supplemental material 

Figure S1

Table S1

### Study design 

For IV dosing, each vial of mepolizumab was reconstituted in 5 mL of sterile water for injection (WFI) and then diluted in a saline bag for infusion. Mepolizumab IV was administered for ~ 30 – 60 minutes. For the SC dosing, each vial of mepolizumab was reconstituted in 2 mL of sterile WFI. All subjects receiving SC injections received two injections of the same volume, allowing single blinding (subject) on these three arms. For consistency and as internal control, (active) mepolizumab was administered in the right upper arm for the medium and low doses (i.e., 125 and 12.5 mg, respectively) and the placebo injection was administered in the left upper arm. The high dose (i.e., 250 mg) of mepolizumab was administered in both the right and left upper arms. Sterile WFI was initially used for the placebo injection in the left arm for the 125 and 12.5 mg SC doses and for the dilution steps in the 12.5 mg dose. WFI was changed to saline after some subjects reported injection site pain. 

### Study population 

Current smokers (within 6 months of screening) or subjects with a smoking history of > 10 pack-years; subjects with an exacerbation or respiratory tract infection within 6 weeks prior to screening; a clinically important lung condition other than asthma (e.g., emphysema or chronic bronchitis (i.e., chronic obstructive pulmonary disease) or a history of lung cancer); a previous history of cancer in remission for less than 5 years prior to screening (except for localized carcinoma of the skin that had been resected for cure) or current malignancy were excluded. Subjects were also excluded if they displayed QT interval abnormalities; had clinically significant systemic abnormalities uncontrolled with standard treatment; had a positive pre-study hepatitis B surface antigen or positive hepatitis C antibody result within 3 months of screening; had known immunodeficiency; had a positive pre-study drug/alcohol test at screening; had been exposed to live vaccine within the 4 weeks prior to screening; and had recently been using prescription or non-prescription drugs (including vitamins, herbal and dietary supplements). 

### Analytical methods supporting information 

**Pharmacokinetic assay**

Blood samples (~ 4 mL) for determination of plasma concentrations of mepolizumab were collected into plastic Lithium Heparin tubes and immediately placed on ice. The plasma was separated by refrigerated centrifugation 4 °C (20 minutes at ~ 1,500 g or 3,000 rpm). Plasma samples were transferred to appropriately labelled polypropylene tubes, frozen on solid carbon dioxide (Dry Ice) and stored at the investigator site at ~ –20 °C until shipment for analysis at Alliance Pharma Inc. (Malvern, PA, USA). If samples could not be frozen in solid carbon dioxide, they were placed directly in the –20 °C freezer. Both immunoassay methods were shown to be sensitive, selective, reproducible and accurate for the determination of mepolizumab in human plasma, with an analytical range of 50 – 5,000 ng/mL. There was no change in the overall performance of the analytical method as a result of the capture reagent substitution between the 2 immunoassays used. The precision and accuracy of both assays as determined by the percent coefficient of variation or percent bias was ≤ 9.1% and ± 7.8% respectively at all known quality control concentrations measured within the analytical range of both methods. 

**Immunogenicity assay**

Approximately 6.0 mL of whole blood was collected into serum-separator tubes with clotting-activator (e.g., BD red or marble top tubes). The tubes were maintained at ambient temperature for 1 hour to allow for complete clotting prior to centrifugation. Once a sample had completely clotted, it was centrifuged (room temperature) for 10 minutes at 2,000 g. Serum was aliquoted to 3 cryo vials (at equal volume). These filled cryo vials were immediately frozen at –70 °C to –80 °C and stored at this temperature until shipment for analysis by Clinical Immunology, King of Prussia, PA (USA). 

For the validation of the ADA assay for the presence of anti-mepolizumab antibodies analysis, the following parameters were evaluated using purified rabbit anti-drug antibodies: assay precision (< 25% CV), screening and confirmation cut points, specificity, drug interference, sensitivity (1.03 ng/mL), and stability. For the detection, test samples were mixed with an anti-IL-5 blocking antibody, and then overnight with biotin and Sulfo-TAG drug conjugates to allow bridge formation. ADAs binding to both conjugates and the streptavidin-coated Meso-Scale Discovery (MSD) plate resulted in an electrochemiluminescent signal directly proportional to the amount of ADAs in the sample. 

**Free and total IL-5 assays**

Approximately 3.0 mL of whole blood was collected into serum separator tubes with clotting activator (e.g., BD red or marble top tubes). The tubes were maintained at ambient temperature for 1 hour to allow for complete clotting prior to centrifugation. Once the sample had completely clotted, it was centrifuged at 15 – 25 °C for 10 minutes at 2,000 g. Serum was aliquoted to 2 cryo vials (at equal volume). These filled cryo vials were immediately frozen at –70 °C to –80 °C and stored at this temperature until shipment for analysis by Clinical Immunology, King of Prussia, PA (USA). 

The total IL-5 assay used an anti-IL-5 monoclonal antibody for capture and a biotinylated anti-IL-5 monoclonal antibody for detection, and was designed to provide accurate IL-5 quantification in the presence of excess mepolizumab. Serum total IL-5 levels were back-calculated against a calibration curve of GSK-provided IL-5. 

The free IL-5 assay used mepolizumab for capture and a rat anti-IL-5/Sulfo-TAG labeled goat anti-rat antibody combination for detection, and was thus designed to capture IL-5 that is not complexed with mepolizumab. 

Validation parameters included evaluation of precision, accuracy/matrix, specificity, linearity and analyte stability. All evaluated parameters were within acceptance criteria of 25% CV’s or less and 25% RE’s or less. The methods were considered “fit-for-purpose” and implemented into clinical testing. 

### Derived PD parameters supporting information 

The following pharmacodynamic parameters for either blood or sputum data were derived as follows:

- AUEC_eos_ for an individual subject: AUEC_eos(t0-tn) _= sum (AUECt_0_-t_1_, … AUECt_n-1_-t_n_)

where:

- AUECt_i-1_-t_i_ = 1/2 (e_i-1_+ e_i_)(t_i_-t_i-1_)
- e_i-1_ and e_i_ are two adjacent eosinophil measures
- t_i-1_ and t_i_ are two adjacent time points.

- Area above the eosinophil time curve to day 84 for an individual subject as a proportion of the total area under the baseline eosinophils to day 84, (i.e., the proportional inhibition of the total area under the baseline eosinophils to day 84): Proportional inhibition AUEC_eos(0-Day84)_ = 1 – {AUEC_eos(0-Day84*)_ /[Baseline eosinophils × t_Day84*_]}.
- Weighted mean eosinophils (day 0 – 84) for an individual subject: wmean_eos(0-day 84)_ = (AUEC_eos(0-Day84*)_)/(t_Day84*_)    *day 84 visit or last day with available eosinophil data prior to day 84.

### Population pharmacokinetic model 

The IV and SC models, using the ADVAN 3 and ADVAN 4 subroutine in NONMEM version 7, respectively, were parameterized in terms of a combination of macro and micro constants with estimation of between-subject variability; residual error was modeled by an exponential component. In the SC model, the population estimates for the inter-compartment rate constant parameters were fixed to the values obtained from the IV population PK model. The first order conditional estimation (FOCE) method with interaction was used. 

Exposure over the dosing interval AUC_(0–τ),_ C_max_, and t_max_ were estimated by integration method using the ADVAN 6 subroutine. 

### Population pharmacokinetic/pharmacodynamic model 

Equation for the indirect response PKPD model: 

Where:

- R is the Eosinophil Count (K_in_/K_out_ is the baseline count)
- dR/dT is the rate of change in eosinophils over time
- K_in_ is the rate of production of eosinophils
- K_out_ is the per-capita rate of elimination of eosinophils
- Baseline is equal to K_in_/K_out_
- Imax is the maximal effect (fold reduction in eosinophils)
- r is the Hill slope of the inhibitory curve
- IC_50_ is the concentration associated with 50% of maximal effect
- Cp is the individual predicted mepolizumab plasma concentration at time t.

The PKPD model used the ADVAN 6 subroutine in NONMEM 7 with estimation of between-subject variability; residual error was modeled by an exponential component. The FOCE method with interaction was used. 

### Safety 

Seven subjects reported a total of eight local injection site reactions. Six of the seven subjects reported pain after receiving either mepolizumab diluted with WFI or WFI as placebo. None of these subjects reported a recurrence of pain on subsequent dosing when mepolizumab was diluted with an isotonic solution of saline or they received saline as placebo. This suggests that the hypotonic WFI was the cause of the initial reports of pain in these subjects. There were no reports of severe acute anaphylactic reactions during the study. No clinically relevant trends were observed in laboratory, vital signs, electrocardiogram (ECG), or other safety data. A summary of the most frequent on-therapy adverse events are given in Supporting Table S1. 

## 

**Equation.**
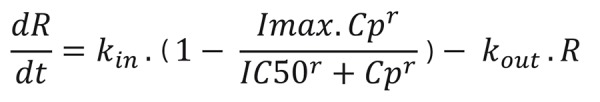
Equation

**Table 1.** Subject demographic and baseline characteristics.

	Mepo SC 12.5 mg n = 21	Mepo SC 125 mg n = 15	Mepo SC 250 mg n = 23	Mepo IV 75 mg n = 11	Total n = 70
Age in years, mean (range)	43.1 (18 – 60)	37.0 (18 – 63)	43.9 (18 – 61)	44.8 (20 – 60)	42.3 (18 – 63)
Sex, n (%)					
Female:	13 (62)	5 (33)	14 (61)	5 (45)	37 (53)
Race, n (%)					
White	20 (95)	14 (93)	21 (91)	11 (100)	66 (94)
African American/African heritage	1 (5)	0	1 (4)	0	2 (3)
Asian	0	1 (7)	1 (4)	0	2 (3)
Ethnicity, n (%)					
Hispanic or Latino:	2 (10)	0	0	0	2 (3)
Not Hispanic	19 (90)	15 (100)	23 (100)	11 (100)	68 (97)
Weight in kg, Mean (range)	73.86 (46.0 – 114.6)	72.81 (42.5 – 101.0)	74.81 (42.0 – 160.0)	83.85 (51.0 – 120.4)	75.52 (42.0 – 160.0)
Body mass index in kg/m^2^, Mean (range)	25.24 (17.2 – 37.0)	24.41 (17.2 – 33.4)	26.00 (16.8 – 51.7)	27.80 (18.6 – 35.3)	25.71 (16.8 – 51.7)
Baseline blood eosinophils in GI/L, Mean (range)	0.583 (0.19 – 1.70)	0.461 (0.15 – 1.18)	0.586 (0.22 – 2.42)	0.348 (0.19 – 0.55)	–
Baseline sputum eosinophils in %, Mean (range)	22.97 (0.75 – 69.25)	10.94 (1.75 – 53.25)	20.88 (0.50–71.5)	6.78 (0 – 12.75)	–
Baseline FEV1 in L, Mean (range)	2.375 (0.99 – 3.72)	2.593 (1.82 – 3.70)	2.102 (1.16 – 3.83)	2.411 (1.43 – 3.80)	–
Baseline FVC in L, Mean (range)	3.468 (1.67 – 5.92)	3.751 (2.48 – 5.31)	3.275 (1.81 – 5.14)	3.623 (2.42 – 6.11)	–
Total IL-5 at baseline, n (%) (LLQ = 7.81 pg/mL)	3 (14)	2 (13)	1 (4)	2 (18)	–
Free IL-5 at baseline, n (%) (LLQ = 3.91 pg/mL)	1 (5)	1 (7)	2 (9)	0 (0)	–

FEV1 = forced expiratory volume in 1 second; FVC = forced vital capacity; IL = interleukin; IV = intravenous; LLQ = lower limit of quantification; SC = subcutaneous.

**Table 2.** Mepolizumab population pharmacokinetic parameter estimates from the intravenous and subcutaneous population pharmacokinetic analyses.

Parameters (IV)	Estimate (95% CI)	BSV
CL (L/day)	0.210 (0.189 – 0.232)	23.3%
Vc (L)	3.60 (3.19 – 4.05)	17.2%
Vp (L)	3.56	NA
Kcp (/day)	0.280 (0.214 – 0.367)	60.6%
Kpc (/day)	0.283 (0.233 – 0.344)	NA
RESIDUAL	0.214 (0.142 – 0.286)	WSB (95% CI) 21.6% (14.3 – 29.2)
Parameters (SC)		
CL/F (L/day)	0.310 (0.275 – 0.349)	57.7%
Vc/F (L)	4.57 (4.02 – 5.20)	59.3%
Vp/F (L)	4.53	NA
Kcp (/day)	0.280 (fixed)	NA
Kpc (/day)	0.283 (fixed)	NA
KA (/day)	0.194 (0.155 – 0.242)	87.2%
RESIDUAL	0.333 (0.279 – 0.387)	WSB (95% CI) 34.2% (28.5 – 40.1)

CL = plasma clearance; Vc = volume of the central compartment; Vp = volume of the peripheral compartment; Kcp = rate constant (from central to peripheral compartment); Kpc = rate constant (from peripheral to central compartment); CL/F = apparent clearance; Vc/F = apparent volume of the central compartment; Vp/F= apparent volume of the peripheral compartment; KA = absorption rate constant; NA = not applicable; CI = confidence interval; BSV = between-subject variability; WSB = within-subject variability. Vp, Vp/F and WSB (95% CI) were calculated post-hoc. Vp and Vp/F estimates are derived from their composite parameters. Residual error model: Y = F+F×θ_i_×(ε_i_) with ε_i_ = 1; 95% CI = estimate ± 1.96 × SE; SE = standard error; %CV = 100 × SQRT(exp(x)-1) with x = ETA or x = (θ_i_×(ε_i_))^2^; ETA = variance.

**Table 3.** Mepolizumab population pharmacodynamic parameter estimates from the population pharmacokinetic/pharmacodynamic analysis.

Parameters	Estimate (95% CI)	BSV
KRO (GI/L)	0.710 (0.642 – 0.784)	38.5%
KOUT (/day)	0.414 (0.297 – 0.578)	NA
IC_50_ (ng/mL)	1261 (878 – 1813)	NA
IMAX	0.928 (0.875 – 0.959)	NA
BL covariate on KRO	0.701 (0.544 – 0.858)	NA
RESIDUAL	0.471 (0.419 – 0.518)	WSB (95% CI) 49.9% (43.8 – 55.5)

KRO = blood eosinophils baseline; KOUT = blood eosinophils rate of elimination; IC_50_ = concentration inducing 50% of the maximum inhibitory effect; IMAX = maximum inhibitory effect; BL = baseline, BSV = between-subject variability; WSB = within-subject variability; NA = not applicable. WSB (95% CI) was calculated post-hoc. Residual error model: Y = F×exp(ε_i_); 95% CI = estimate ± 1.96 × SE; SE = standard error; %CV = 100 × SQRT(exp(x)-1) with x = ETA or x = ε_i_; ETA = variance.

**Table 4.** Summary of derived blood and sputum eosinophil parameters by treatment group.

Parameter (unit)	Summary statistics	Mepolizumab dose			
		SC 12.5 mg n = 21	SC 125 mg n = 15	SC 250 mg n = 23	IV 75 mg n = 11
Blood eosinophils					
AUEC_eos(0–day 84)_ (GI.d/L)	n	20	14	21	11
	Geo mean	21.551	7.198	6.381	7.556
	95% CI	15.486 – 29.991	5.290 – 9.796	4.915 – 8.284	5.459 – 10.459
Proportional inhibition AUEC_eos(0–day 84)_	n	20	14	21	11
	Geo mean	0.396	0.743	0.818	0.687
	95% CI	0.263 – 0.596	0.679 – 0.813	0.780 – 0.857	0.602 – 0.784
wmean_eos(0–day 84)_ (GI/L)	n	21	15	23	11
	Geo mean	0.251	0.090	0.083	0.090
	95% CI	0.183 – 0.345	0.066 – 0.121	0.063 – 0.109	0.065 – 0.125
Max_eos_ (GI/L)	n	21	15	23	11
	Geo mean	0.203	0.113	0.082	0.141
	95% CI	0.124 – 0.331	0.079 – 0.162	0.057 – 0.119	0.085 – 0.233
tmax_eos_ (Days)	n	21	15	23	11
	Arithmetic mean	50.0	49.4	47.0	58.8
	95% CI	34.6 – 65.5	34.0 – 64.8	32.0 – 62.0	42.0 – 75.6
Subjects achieving ≥ 50% repletion	n (%)	8 (38)	1 (7)	2 (9)	1 (9)
Sputum eosinophils					
Proportional Inhibition AUEC_speos(0–day 84)_	n	10	5	13	7
	Geo mean	0.315	0.627	0.693	0.690
	95% CI	0.125 – 0.793	0.377 – 1.045	0.562 – 0.856	0.579 – 0.821
wmean_speos(0–day 84)_ (%)	n	16	8	13	7
	Geo mean	7.734	1.368	2.551	1.938
	95% CI	3.914 – 15.283	0.772 – 2.424	1.075 – 6.054	0.580 – 6.473
Max_speos_ (%)	n	15	6	13	7
	Geo mean	0.228	0.025	0.042	0.122
	95% CI	0.112 – 0.465	0.006 – 0.101	0.016 – 0.110	0.032 – 0.466
tmax_speos_ (Days)	n	15	6	13	7
	Arithmetic mean	33.6	43.2	50.6	27.0
	95% CI	20.7 – 46.5	14.4 – 72.0	33.2 – 68.0	6.7 – 47.3

AUEC_eos(0–day 84)_ = area under the absolute blood eosinophil-time curve to day 84 determined using the linear trapezoidal rule, for subset of subjects with blood eosinophil data to day 84; proportional inhibition AUEC_eos(0–day 84)_ = area above the absolute blood eosinophil-time curve to day 84 as a proportion of the total area under the baseline blood eosinophil level to day 84, for subset of subjects with blood eosinophil data to day 84; wmean_eos(0–day 84)_ = weighted mean absolute blood eosinophil levels (day 0 – 84 or last day with available eosinophil data prior to day 84; Max_eos_ = maximum reduction from baseline in blood eosinophils (between day 0 and last quantifiable measurement); tmax_eos_ = time to first occurrence of maximum reduction from baseline in blood eosinophil levels (between day 0 and last quantifiable measurement); proportional inhibition AUEC_speos(0–day 84)_ = area above the percent sputum eosinophil-time curve to day 84 as a proportion of the total area under the baseline percent sputum eosinophil level to day 84; wmean_speos(0–day 84)_ = weighted mean percent sputum eosinophil levels (day 0 – 84 or last day with available eosinophil data prior to day 84; Max_speos_ = maximum reduction from baseline in percent sputum eosinophils; tmax_speos_ = time to maximum reduction in percent sputum eosinophil levels; Geo = geometric; CI = confidence interval.

**Table S1.** Summary of most frequent on-therapy adverse events (any AE that occurred in more than one subject in any one dose group).

n (%)	Mepo SC 12.5 mg n = 21	Mepo SC 125 mg n = 15	Mepo SC 250 mg n = 23	Mepo SC All doses n = 59	Mepo IV 75 mg n = 11
Subjects with any AE(s)	14 (67)	7 (47)	12 (52)	33 (56)	6 (55)
Injection site reaction*	3 (14)	3 (20)	1 (4)	7 (12)	0
Asthma	4 (19)	1 (7)	1 (4)	6 (10)	1 (9)
Nasopharyngitis	3 (14)	1 (7)	2 (9)	6 (10)	1 (9)
Arthralgia	1 (5)	0	0	1 (2)	2 (18)
Cough	2 (10)	0	0	2 (3)	0
Headache	0	0	2 (9)	2 (3)	0

*Six of the 7 subjects experiencing a local injection site reaction reported pain after receiving either mepolizumab diluted with water for injection (WFI) or WFI as placebo. AE = adverse event; SC = subcutaneous; IV = intravenous.
